# Deodorizers and Disinfectants

**Published:** 1871-08

**Authors:** 


### DEODORIZERS AND DISINFECTANTS.

THE former takes away the bad smell for the time being, hence requires repetitions; the latter removes the odor, and arrests that process of change which gives rise to the noisomeness. Dissolve copperas in water, throw it on a heap of carrion, and no smell will be perceptible for a while. Throw over this same carrion a solution made by dissolving a pound of crystallized carbolic acid in a hundred pounds or pints of water, and not only does the smell disappear, but the solution tends to prevent further decay of the decomposing flesh, and if strong enough, the decay will be indefinitely postponed.

But there is a sham deodorization in fashionable life which merits notice. Ill-smelling persons always go into company highly scented with musk, cologne water, or other material; these simply overpower the fetor, they do not absorb or take it away, and every breath of air drawn contains not only the original personal odor but that of the scent besides; in other words is doubly impure. Burning coffee or sugar in the sick-chamber takes no foul odor away, but by its greater intensity prevents the notice of it. The healthiest scent in the world is no scent at all, where the air is so pure that the breathing of it attracts no attention whatever; hence the only perfect deodorizer is perfect cleanliness of person and premises; and it is especially important in the warm summer time to put on clean clothing often; to wash feet and head and hands, arms and armpits every day; to go around and through the premises often, from cellar to attic, and allow no pile of dirt, dry or moist, no offal whatever to remain for a single moment; dampness should be especially guarded against; every room in a house should be thoroughly aired every sunshiny day, the earlier in the morning the better.

The dining-room and parlor should be aired by opening doors and windows at daylight, and closing them within half an hour after sunrise, and then kept moderately dark for the most part; this keeps out the flies, and if judiciously managed the apartments will be found to be comfortably cool during the whole day; at sundown, open the windows and doors again; but if mosquitoes are about, have no lights burning.

### VENTILATING CHAMBERS.

The best management of sleeping rooms in summer is to air them most thoroughly every sunshiny day, and not at all in cloudy or damp weather, for if the windows are opened the bedding will absorb the dampness; keep only inner doors and fireplace open. From ten o'clock until three the windows, doors, and fireplace of sleeping-rooms, in clear sunshiny days, should be kept open; the bedclothes should be spread out, and the beds or mattresses should have the fullest exposure possible by being thrown over the foot rail of the bedstead; at three make up the bed, and adjust the room.

### OPEN WINDOWS AT NIGHT.

Very much has been written on this subject, and written unwisely; the facts are that whoever sleeps uncomfortably cool will get sick. To hoist a window sky-high when the mercury is at zero is an absurdity.

The colder a sleeping apartment is, the more unhealthy does it become, because cold condenses the carbonic acid formed by the breathing of the sleeper. It settles near the floor and is rebreathed, and if in a very condensed form, he will die before the morning. Hence we must be governed by circumstances; the first thing is, you must be comfortably warm during sleep, otherwise you are not refreshed, and inflammation of the lungs may be engendered, and life destroyed within a few days.

An open door and an open fireplace are sufficient for ordinary purposes in very cold weather. When outer windows are opened, it is well to have them down at the top two or three inches, and up at the bottom for the same space.

In miasmatic localities, — and these are along water-courses, beside millponds, marshes, bayous, river bottoms, flat lands and the like, — it is most important, from the first of August until several severe frosts have been noticed, to sleep with all external doors and windows closed, because the cool air of sunset causes the condensation of the poisonous emanations which were caused by the heat of the noonday sun to rise far above the earth; this condensation makes the air "heavy" at sundown, made heavy by the greater solidification of the emanations by cold; and resting on the surface of the earth in their more concentrated and malignant form, they are breathed into the lungs, and swallowed into the stomach, corrupting and poisoning the blood with great rapidity. By daylight these condensations are made so compact by the protracted coolness of the night, that they are too near the surface of the earth to be breathed into the system; but as the sun begins to ascend, these heavy condensations, miasms, begin to rise again to the height of several feet above the ground, and are freely taken into the system by every breath and swallow; hence the hours of sunrise and sunset are the most unhealthful of all the hours of the twenty-four in the localities named; and noontide, when the sun is hottest, is the most healthful portion of the day, because the miasm is so much rarefied that it ascends rapidly to the upper regions.

The general lessons are, 1st. Avoid exposure to the outdoor air in miasmatic localities for the hours including sunrise and sunset. 2d. Have a blazing fire on the hearth of the family room at those hours, to rarefy and send the miasm upwards. 3d. Take breakfast before going out of doors in the morning, and take tea before sundown; then being out after night is not injurious.

